# Climate Change Is Increasing the Risk of the Reemergence of Malaria in Romania

**DOI:** 10.1155/2016/8560519

**Published:** 2016-10-26

**Authors:** Larisa Ivanescu, Ilie Bodale, Simin-Aysel Florescu, Constantin Roman, Dumitru Acatrinei, Liviu Miron

**Affiliations:** ^1^Department of Clinics, Faculty of Veterinary Medicine, “Ion Ionescu de la Brad” University of Agricultural Sciences and Veterinary Medicine, 3 M. Sadoveanu, 700490 Iasi, Romania; ^2^Department of Physics, “Alexandru Ioan Cuza” University, 11 Carol I, 700506 Iasi, Romania; ^3^“Dr. Victor Babes” Infectious and Tropical Diseases Clinical Hospital, 281 Mihai Bravu, District 3, Bucharest, Romania

## Abstract

The climatic modifications lead to global warming; favouring the risk of the appearance and development of diseases are considered until now tropical diseases. Another important factor is the workers' immigration, the economic crisis favouring the passive transmission of new species of* culicidae* from different areas. Malaria is the disease with the widest distribution in the globe. Millions of people are infected every year in Africa, India, South-East Asia, Middle East, and Central and South America, with more than 41% of the global population under the risk of infestation with malaria. The increase of the number of local cases reported in 2007–2011 indicates that the conditions can favour the high local transmission in the affected areas. In the situation presented, the establishment of the level of risk concerning the reemergence of malaria in Romania becomes a priority.

## 1. Introduction

Malaria is a widely spread disease in the tropical and subtropical areas [1].

It is a worldwide spread disease, millions of people being affected by it every year in Africa, India, South-East Asia, the Middle East, and Central and South America, which means that almost 50% of the world's population is at risk of being infected with malaria [2]. Every year, millions of sick people or people carrying the disease travel to malaria-free countries, reintroducing the reemergence risk of this disease. In 2010, Snow and colleagues presented a new cartographic technique that would produce a real image of the spread of malaria worldwide. Only the health reports of 7 out of the 87 endemic countries may be considered rigorous and therefore may be used for the information they provide. There has been a quantification of the anthropic impact on the distribution of malaria in the XXth century, using the geographic information systems and historical maps for the areas with continuous transmission of the disease; this overall estimate shows that malaria prevalence is 50% bigger than that reported by the World Health Organization (WHO) and 200% bigger in the case of the areas outside Africa [3–5]. Climatic factors are directly involved in the geographic distribution and transmission of malaria [6, 7], playing a very important role in the malaria reemergence risk in some parts of the world, where the vectors are present. In 2006 it was proved that an increase of temperatures by 0.5°C in the mountainous areas of Africa would lead to a 100% increase of the mosquito population, which would result in a rapid spread of the disease in this area with sporadic cases as well [8].

In 2011, there have been 40 cases of people infected with* Plasmodium vivax* reported in Greece, in five different districts, in the case of patients with no history of travelling to a malaria-endemic area. Currently, there are preventive measures in place, on a seasonal (spring–autumn) basis, in areas which do not present any risk of spreading malaria in other countries, the affected areas being agricultural and not tourist ones (epidemiological update: malaria in Greece, 20th July 2012, European Centre for Disease Prevention and Control). Some cases of autochthonous malaria have been reported in Germany as well [9]. In the last 10 years, the sporadic malaria transmission has been reported in many countries of Europe: Bulgaria, France, Germany, Greece, Italy, and Spain. Between 21st May and 5th December 2011, in Greece, there have been reported 63 cases of malaria, caused by* Plasmodium vivax*. Based on all available information, it appears that the malaria transmission in Greece would be a result of the annual introduction of the parasite in the country, through the presence of immigrants [10]. Malaria is rarely diagnosed in Europe, but it represents a medical emergency. Malaria was eradicated in Europe, with the exception of Azerbaijan, Georgia, Kyrgyzstan, Tajikistan, and Turkey [11].

Malaria was eradicated in Romania in 1965, being considered in 1967 a malaria-free area by the World Health Organization (WHO) [12]. The presence of the* Anopheles* vector, belonging to the* Anopheles maculipennis* complex, and the existence of the malaria agent in nature with imported malaria cases, as well as the increase in temperatures, are favourable factors for the reemergence of malaria in Romania [13, 14]. All cases of malaria diagnosed in Romania have been “imported,” increasing continuously because of the development of tourism and the labour market in malaria-endemic areas [15–17]. The studies carried out by us in Iaşi, Romania, reported the presence of five species of mosquitoes belonging to the* Anopheles maculipennis* complex:* A. atroparvus, A. melanoon, A. maculipennis*,* A. labranchiae, and A. messeae*, species incriminated in the transmission of malaria in Europe.* A. labranchiae* was first reported in Romania, being considered one of the main malaria vectors in Europe, its identification being done using the PCR technique and sequencing, being able to also find the fourth-stage larvae, which indicates that the species adapted to the climate specific to the area [17].

The presence of this species in Romania shows on the one hand the existence of favourable climatic factors, caused by global warming, and on the other hand the increase in people's travels, thus suspecting that the species was “imported” from Italy, Sicily, where many Romanians are working [18, 19].

## 2. Material and Method

The study carried out had the purpose of establishing the existence of the risk of malaria reemergence in Romania on the basis of the coexistence of the most important factors: the presence of the pathogen causing malaria and the existence of favourable climatic factors for the development of the* Anopheles* vector and its existence in nature.

The work of establishing the risk of malaria reemergence in Romania based on the existence of the malaria agent in nature was done based on the analysis of the data concerning the malaria cases diagnosed in Romania, coming from four diagnosis centres from the country: the Public Health District Authority of Iaşi, Infectious Diseases Hospital of Iaşi, Cantacuzino National Institute of Research, and “Victor Babeş” Infectious and Tropical Diseases Hospital. We must note that in Romania there is not a common, centralised database, where all diagnosed malaria cases are reported; therefore all information presented may not paint 100% the real situation. The cases were distributed and analysed over the course of 7 years, according to the following criteria: age, profession, the strain of* Plasmodium* diagnosed, and the continent where the patients acquired the infection.

Having in mind the purpose of monitoring the influence of the environmental factors on the life cycle of the mosquito vector and on the development of the malaria pathogen inside the vector, we did an analysis of the temperatures recorded between 1961 and 2014, at five weather stations in the country (from Iaşi, Cluj-Napoca, Arad, Bucharest, and Constanţa), all information being granted by the National Weather Administration. There was also a weather forecast made for the year 2030, in order to anticipate the possibility of the malaria reemergence risk in Romania, thus associating with this one the other two essential factors: the mosquito vector and the existence of the malaria pathogen in nature.

The mathematical model we proposed, called ET30, is based on the construction of a nonlinear mathematical function (Lagrange polynomial) which allows us to estimate the average temperature values in the next years, avoiding the weather forecast with fixed point, which is very difficult to do for a short period of time (days) and practically impossible to realise for a longer period of time, such as decades [20]. Using this type of mathematical algorithm allows us to find a function *F*, through the interpolation of the experimental measurements, which would estimate the average temperature of each month for the year 2030, according to the climate tendency recorded in the last five decades. From a mathematical point of view, the experimental measurements are the solutions to an unknown function *f*, defined as a table function, represented by the values of air temperature, at the height of 2 m above the ground (*T*_January1961_, *T*_February1962_,…, *T*_December2013_), recorded between January 1961 and December 2014, called interpolation points or interpolation nodes. The interpolation function *F* must have the value of the unknown function *f*, in the interpolation points *T*_*i*_, must be a continuous function along the period of time during which the measurements were taken (January 1961–December 2014), and must also be a Lagrange polynomial nonlinear function. Having in mind the purpose of increasing the estimation accuracy of temperatures at ground level, in the next decades, we have used the mathematical model for three sets of temperature values recorded between 1st of January 1961 and December 2014: the first set of values is represented by the average values of the air temperature, during the day, calculated with the arithmetic mean of the values recorded in a day, the second set of values is represented by the average of monthly temperatures, calculated with the arithmetic mean of the values recorded in a month, during the daytime, and the last set of values is represented by the average of temperatures recorded during five consecutive years, for the same month. We have proposed this estimation method with the purpose of reducing the daily fluctuations. In order to see the reliability of the model, it was tested for the years 1991 and 1992, by having built a model, in the same way as was previously presented, for the values recorded between 1st of January 1961 and 31st December 1990 and another model for estimating the values for the years 2011, 2012, and 2013, using the values recorded between 1st of January 1961 and 31st December 2010. The program sequences of model ET30 were calculated using the programming language FORTRAN, the data processing being done using the OringiPro.

## 3. Results and Discussion 

In Romania, the number of malaria cases grew during the war; the strains of* Pl. falciparum* introduced on this occasion increased the gravity of the cases and the mortality rate. The situation was quite drastic in the county of Tulcea, in 1946. In some towns or villages, the malaria infection affected 70% of the population.

Between 1947 and 1948, the fight for the eradication of malaria was at its peak, having used DDT (dichlorodiphenyltrichloroethane), the results however not being exactly in accordance with what had been targeted, namely, complete eradication of the disease. The campaign against malaria continued, starting from 1955 with eradication plans for the disease. The last case was diagnosed locally in 1961. In 1965, the malaria eradication campaign ended, Romania being declared a malaria-free country by the World Health Organization (WHO) in 1967.

Starting from 1967 (the year starting which we had all information regarding the diagnosed cases of malaria) and until 2014, there have been 814 cases diagnosed in Romania, all of them being “imported.” Regarding the details about the profession, the age, the strain of* Plasmodium* diagnosed, and the continent where the patients acquired the infection, we had such information only for the last 7 years. In 2008, there were 13 cases diagnosed, in 2009 there were 12 cases diagnosed, in 2010 there were 19 cases diagnosed, in 2011 there were 42 cases diagnosed, in 2012 there were 17 cases diagnosed, in 2013 there were 31 cases diagnosed, and in 2014 there were 39 cases diagnosed, in total being 173 cases, which represent 14% of all diagnosed cases between 1976 and 2014.

**Figure 1:**
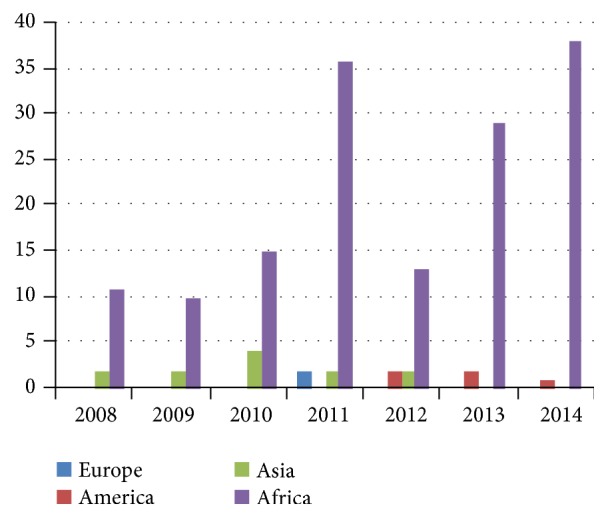
The distribution of malaria cases according to the continent where the infection occurred.

Out of the total number of cases diagnosed, 88% of them occurred on the continent of Africa, the continent of Asia following close behind, with 8% of the cases (Figure 1). In 2011, there were 2 cases recorded in Greece (a malaria-free country), these being considered autochthonous cases, which indicates that all three conditions of the vector-borne disease were met: the presence of the* Anopheles* vector, the presence of the* Plasmodium* pathogen, and the existence of favourable climatic factors.

**Figure 2:**
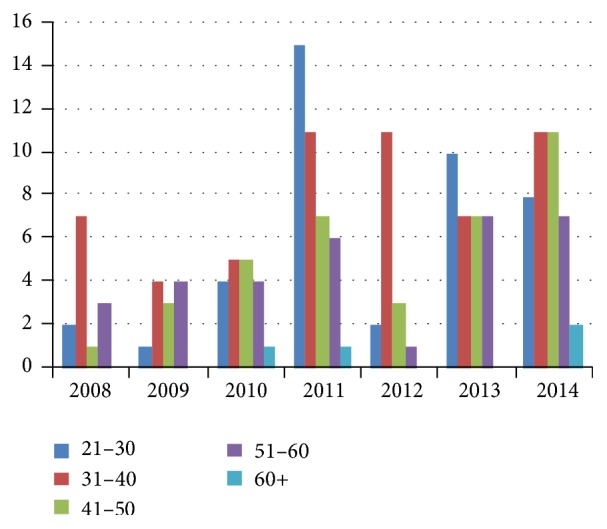
The distribution of malaria cases according to the age factor.

Out of the total malaria cases recorded, 59% were diagnosed in people over 40 years (Figure 2), representing the category of population with the highest labour force level.

**Figure 3:**
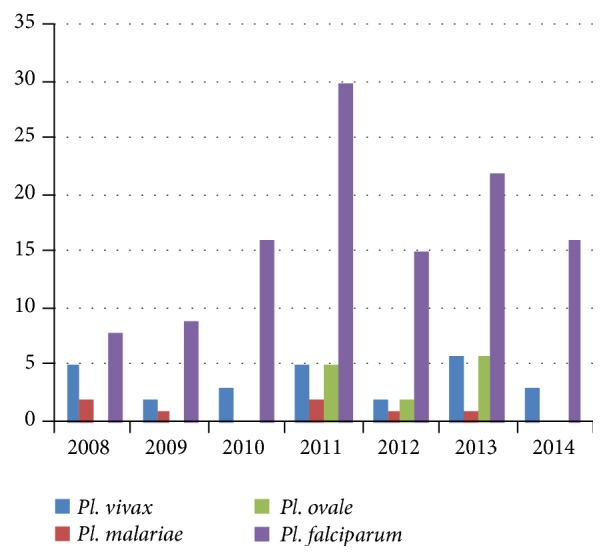
The distribution of malaria cases according to the strain of* Plasmodium* responsible for the infection.

*Plasmodium falciparum *was diagnosed in 75% of the cases, being responsible for the infection with cerebral malaria, all the more dangerous for those living in countries that were not yet affected, meaning that the degree of susceptibility was quite high.* Plasmodium vivax *was responsible for 13% of the cases, being a very dangerous strain, along with the strains of* Pl. ovale* and* Pl. malariae* (Figure 3), because of the relapse that may occur even 10 years after the infection, the parasite persisting in the patient's liver, in a dormant, hypnozoite stage. The diagnosis was made using multiple techniques: microscopic examination of thick blood smear, rapid diagnostic test, and, in some cases, the PCR test. There is no centralised database in Romania, so many of the patients who were diagnosed with malaria never returned for regular checkups, constituting a real danger in the case of relapse.

**Figure 4:**
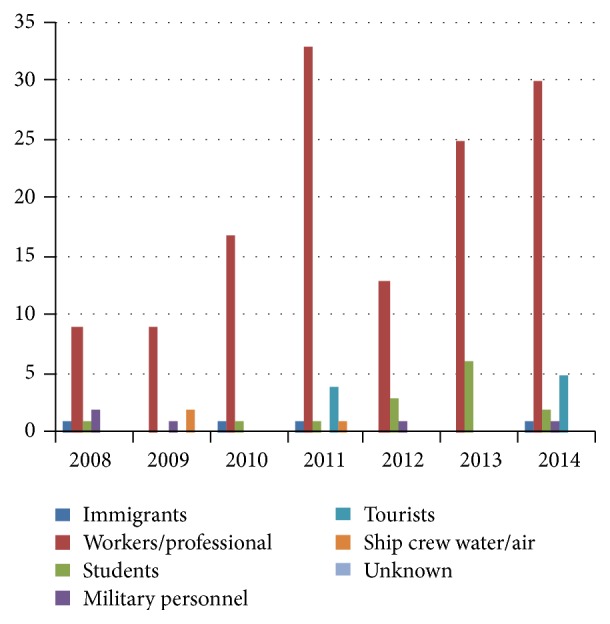
The distribution of malaria cases according to the travel purposes.

In the last 7 years, the labour force was the main reason for travelling, generating 80% of the cases of malaria recorded in Romania, being associated with the onset of the financial crisis of 2007 as well, forcing the population to look for a source of income in places which had not been of any financial interest until that moment (Figure 4). Until 2007, 70% of the total number of diagnosed cases were in the case of people working as sailors. Following close behind, with a percentage of 9%, are students, noting as well an increase of exchange programs and of mobilities between universities on different continents. The majority of people who were diagnosed have not taken any prophylactic medication, have not received any instructions from a doctor before leaving the country, and also did not have any knowledge of the possible dangers existing in tropical areas (the majority of people not having a higher education degree).

**Figure 5:**
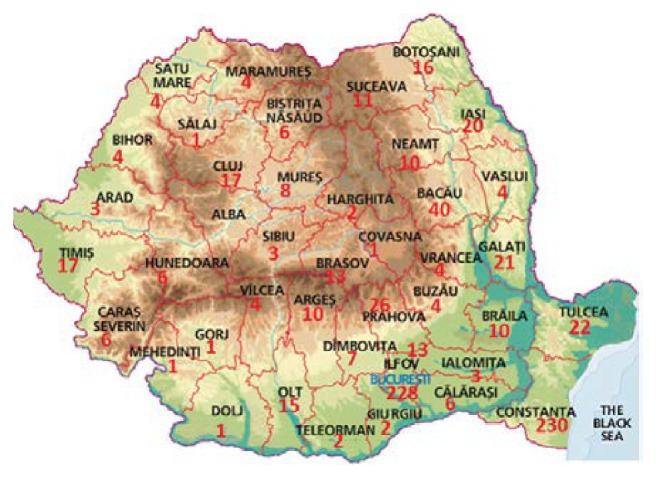
The distribution of malaria cases recorded in Romania between 1976 and 2014.

The map shown in Figure 5 indicates a higher presence of malaria cases in the North-East and in the South-East parts of the country, areas with a low financial potential and with a high unemployment rate, starting from 2007, after the onset of the financial crisis. Bucharest (the capital city) and Constanţa (maritime city) have been areas of great interest until 2007, when over 70% of the diagnosed malaria cases were recorded in the case of people working as sailors, after this year the number of cases dropping considerably in these areas, only 13% of malaria cases being recorded.

Using the mathematical model ET30, we realised a temperature curve starting from 1961, until 2014, indicating a constant increase of temperatures by 1.3°C, compared with the malaria eradication period in the county of Iaşi (Table 1).

**Figure 6:**
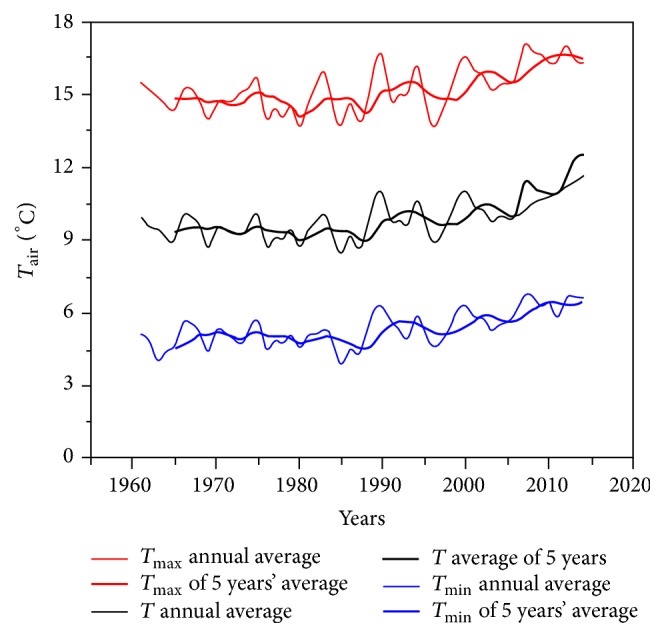
The evolution of the annual maximum temperature, the average annual temperature, and the annual minimum temperature between 1st January 1961 and 31st December 2014, also calculated for five consecutive years.

**Figure 7:**
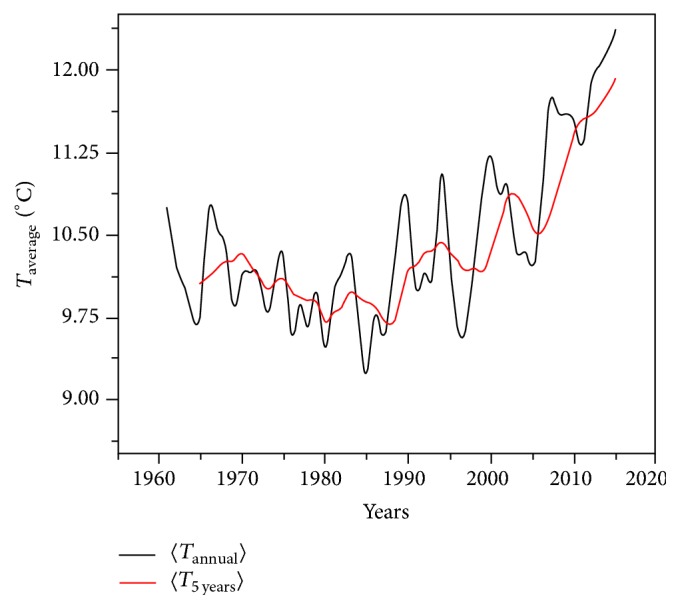
The average annual temperature and the average temperature calculated for five consecutive years, both recorded between 1st January 1961 and 31st December 2010, at 5 weather stations in the country.

The temperatures calculated at five weather stations in the country (Iaşi, Bucharest, Arad, Cluj-Napoca, and Constanţa) also show an increase of temperatures, the total being of 0.72°C (Figures 6 and 7).

The second column indicates the increase of temperatures in 2014 (the average for 10 years, 2004–2014), compared with the period in which malaria was eradicated (1961–1970).

The third column indicates the increase of temperatures in 2014, compared with the 60s, for the average calculated from the data provided by the five weather stations in Romania (Iaşi, Bucharest, Arad, Cluj-Napoca, and Constanţa) (Table 1).

**Figure 8:**
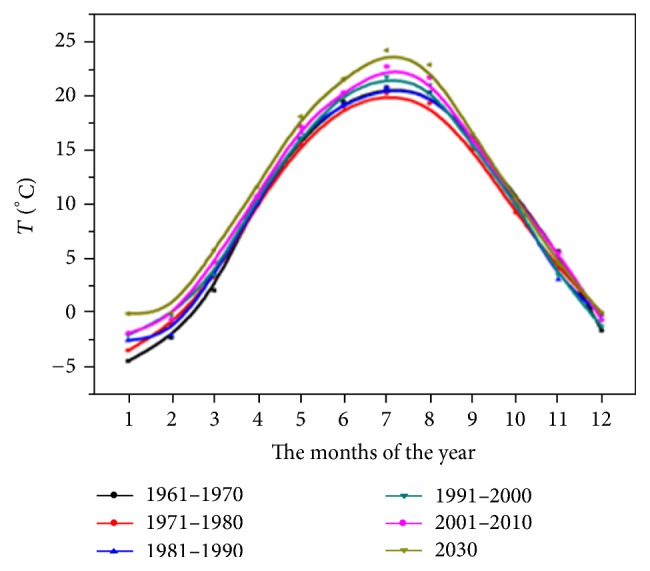
The monthly average temperatures calculated according to decades, recorded at the weather station in Iaşi, between 1961 and 2014, and estimated using the mathematical model ET30 for the year 2030, for each of the twelve months.

Doing an extrapolation of the evolution of temperatures in 2030 (Figure 8), we can see a slight increase of temperatures by an average of 24°C in 2030, which may ensure a favourable climate for the development of Culicidae, the optimal temperatures for development being between 23 and 25°C.

*Anopheles maculipennis* is a species widely spread across the country, near forests and on river valleys, the female being zoophilic and occasionally anthropophilic.

*Anopheles messeae *is spread in all the lowland plains in the country and in places with stagnant waters; it is predominantly zoophilic but may also feed off humans.* Anopheles messeae *may transmit malaria in temperatures even below 4°C, in which case it takes 44 days for the mosquito to become infected. This is how the cases in Scotland and Norway may be explained. In Great Britain,* Anopheles messeae *and* Anopheles atroparvus *are responsible for the cases signaled by Knottnerus [21].* Anopheles atroparvus *is spread all over the country, in areas with (more or less) salt waters, the female being predominantly anthropophilic.* Anopheles atroparvus *is active in temperatures starting from 15°C, when it may constitute a risk in transmitting malaria.

*Anopheles melanoon *may be found near fresh waters, marshland areas, stagnant waters covering large areas, the banks of rivers and lakes, ponds, and swimming pools [12].

In Europe and in the Middle East, the transmission of malaria is very low or absent, but there have been some species of the genus* Anopheles* which were considered vectors of malaria:* A. atroparvus*,* A. labranchiae, A. messeae, A. sacharovi, A. sergentii, and A. superpictus* [22].

**Figure 9:**
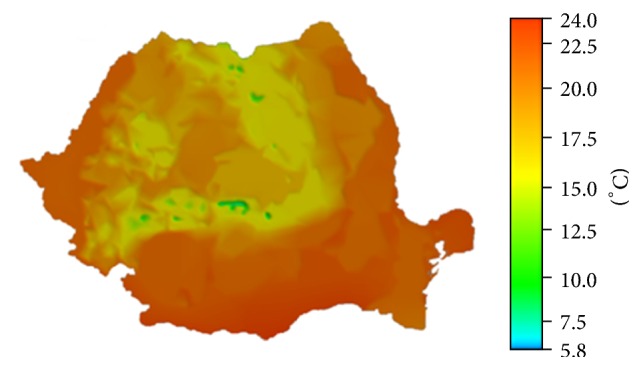
The map of the average temperatures calculated for the month of July in the weather stations of the National Weather Administration network of Romania, for the year 2014.

The illustration of the temperature gradient on the map representing the temperatures in Romania in the month of July was realised using the software ArcGis. For the month of July of the year 2030, we can observe a slight increase in temperatures by 0.9°C (Figure 9).

**Figure 10:**
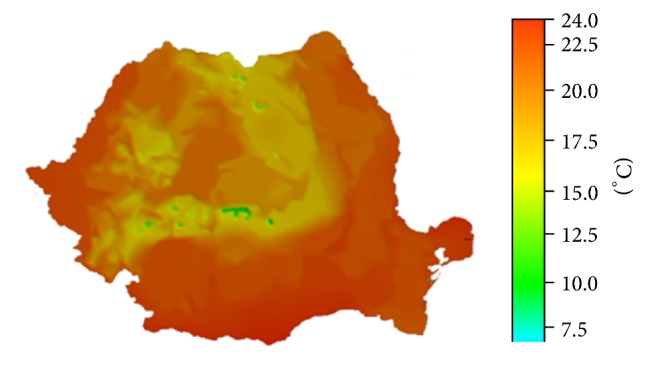
The map of the estimated temperatures for July, for the year 2030, obtained using the model ET30.

The estimations obtained by using the model ET30 (slight warming by 0.8°C, Figure 10), which show an increase in temperatures manifesting in the metropolitan area of Iaşi, keep the same tendency like the forecasts regarding the weather at a global level, done by some of the most prestigious research institutes in the world, such as NIES (the National Institute for Environmental Studies in Japan), CCCma (the Canadian Centre for Climate Modelling and Analysis), CSIRO (the Commonwealth Scientific and Industrial Research Organisation in Australia), HCCPR (Hadley Centre for Climate Prediction and Research in the United Kingdom), MPIM (Max-Planck-Institut für Meteorologie), and NCAR (the National Center for Atmospheric Research in the USA). These institutes provide data which show an increase of temperatures at a global level by 0.8–1.7°C, until the year 2030.

## 4. Conclusions

Taking into account the coexistence of the two factors that are at the basis of the emergence or reemergence of a vector-borne disease, namely, the presence of the malaria agent in nature, as well as the existence of favourable climatic factors for the development of the* Anopheles* vector and of the parasite inside of it, the risk of malaria reemergence in Romania may become a problem of major interest.

In Romania, there is not a common, centralised database, where all diagnosed malaria cases are reported and also, there is no record being kept; therefore, in many cases there is the risk of having a relapse. Studies regarding the “import” of malaria in Romania have been carried out by researchers such as Neghina et al. in 2008 and 2011, the result being the same: difficulty in diagnosing the disease and in managing the important cases. The annual epidemiological report 2014, emerging and vector-borne diseases, keeps the countries which are part of the European Union (EU) and the European Economic Area (EEA) updated on the situation concerning malaria, which seems to have remained stable, occurring in around one case in every 100.000 inhabitants, registering a slight decrease in 2012, in comparison with 2011 and 2010. 99% of the cases (in the case of which the origin of the disease is specified) are “imported”; these cases occurred in countries belonging to the EU and EEA (European Economic Area), which have strong traditional connections with endemic areas. In Greece, there were some autochthonous cases recorded in 2012 but less than those recorded in 2011. The autochthonous transmission of the disease in the EU remains a possibility and therefore the emphasis is placed on the necessity of having medical supervision for the important cases, training medical staff, educating the population, and improving the access to health care services for migrants. In 2012, there have been 5161 confirmed malaria cases in 25 countries from the EU and in one from Continental Europe. 85% of the cases have been reported by the following five countries: France, Great Britain, Germany, Spain, and Belgium. Most of the confirmed cases have been reported by the United Kingdom, Belgium, Ireland, and Luxembourg.

Twenty-six cases have been identified as being autochthonous, out of which twenty-two were from Greece, three were from Belgium, and one was from France. Therefore, some essential measures need to be put in place in order to fight against malaria and to maintain the status of malaria-free country:

- investments in training medical staff and supporting specializations in tropical diseases;
- training medical entomologists, who are very much absent;
- creating a database in order to keep a common record of all the malaria cases recorded at a national level, on Romania's territory;
- following every malaria case diagnosed, through regular medical checkups, especially in the case of cases infected with the strain of* Plasmodium*, which forms hypnozoites in the liver, presenting the risk of relapsing;
- providing hospitals with various drugs, specific for malaria treatment, according to the area of infestation;
- keeping an open communication between tourism companies and hospitals, in order to be able to instruct people on the protection measures, in case they travel to tropical areas.

Keeping the status of malaria-free country becomes a growingly serious mission not only for Romania, but also for other countries in Europe.

With every passing year there is an increase in the number of “imported” malaria cases, which proves the lack of information among the population regarding the risk of contracting the disease and the prophylactic measures and treatment needed.

Climatic factors play a very important role and our studies have shown a favourable evolution in this respect, leading to the existence of the emergence or reemergence of some diseases on Romania's territory.

## Figures and Tables

**Table 1:** The increase of current temperatures according to seasons, compared with the malaria eradication period.

Season	(2004–2014) (1961–1970) Iaşi (°C)	(2004–2014) (1961–1970) Romania (°C)
Winter	1.2	1.4
Spring	1.3	0.8
Summer	1.7	0.9
Autumn	0.2	−0.2
Total	1.1	0.725

+ indicates an increase in temperatures.

− indicates a decrease in temperatures.
